# Psychiatric disorders from EEG signals through deep learning models

**DOI:** 10.1016/j.ibneur.2024.09.003

**Published:** 2024-09-24

**Authors:** Zaeem Ahmed, Aamir Wali, Saman Shahid, Shahid Zikria, Jawad Rasheed, Tunc Asuroglu

**Affiliations:** a Department of Data Sciences, National University of Computer & Emerging Sciences (NUCES), FAST Lahore Campus, Punjab, Pakistan; b Department of Sciences & Humanities, National University of Computer & Emerging Sciences (NUCES), FAST Lahore Campus, Punjab, Pakistan; c Department of Computer Science, Information Technology University (ITU), Lahore, Pakistan; d Department of Computer Engineering, Istanbul Sabahattin Zaim University, Istanbul 34303, Turkey; e Department of Software Engineering, Istanbul Nisantasi University, Istanbul 34398, Turkey; f Faculty of Medicine and Health Technology, Tampere University, Tampere 33720, Finland; g VTT Technical Research Centre of Finland, Tampere 33101, Finland

**Keywords:** Psychiatric Disorders Diagnosis, CNN-LSTM, Mental State Classification, Biomarkers for Mental Health, EEG Signal Processing, Neural Network in EEG

## Abstract

Psychiatric disorders present diagnostic challenges due to individuals concealing their genuine emotions, and traditional methods relying on neurophysiological signals have limitations. Our study proposes an improved EEG-based diagnostic model employing Deep Learning (DL) techniques to address this. By experimenting with DL models on EEG data, we aimed to enhance psychiatric disorder diagnosis, offering promising implications for medical advancements. We utilized a dataset of 945 individuals, including 850 patients and 95 healthy subjects, focusing on six main and nine specific disorders. Quantitative EEG data were analyzed during resting states, featuring power spectral density (PSD) and functional connectivity (FC) across various frequency bands. Employing artificial neural networks (ANN), K nearest neighbors (KNN), Long short-term memory (LSTM), bidirectional Long short-term memory (Bi LSTM), and a hybrid CNN-LSTM model, we performed binary classification. Remarkably, all proposed models outperformed previous approaches, with the ANN achieving 96.83 % accuracy for obsessive-compulsive disorder using entire band features. CNN-LSTM attained the same accuracy for adjustment disorder, while KNN and LSTM achieved 98.94 % accuracy for acute stress disorder using specific feature sets. Notably, KNN and Bi-LSTM models reached 97.88 % accuracy for predicting obsessive-compulsive disorder. These findings underscore the potential of EEG as a cost-effective and accessible diagnostic tool for psychiatric disorders, complementing traditional methods like MRI. Our study's advanced DL models show promise in enhancing psychiatric disorder detection and monitoring, with significant implications for clinical application, inspiring hope for improved patient care and outcomes. The potential of EEG as a diagnostic tool for psychiatric disorders is substantial, as it can lead to improved patient care and outcomes in the field of psychiatry.

## Introduction

The study of neurophysiological signals, such as the electroencephalogram (EEG), is beneficial for understanding mental health problems (Katmah et al., 2021, Garc\’\ia-Ponsoda et al., 2023, Saez and Gu, 2023). The brain's electrical activity on EEG signals can be complex and messy. Therefore, more sophisticated machine learning (ML) models can now be used to assess accurately if someone has a psychological disorder identified on the EEG patterns (Divya et al., 2023). Globally, over 450 million people have schizophrenia, bipolar disorder, and depression (Refriza and Samudro, 2021, Shadhin et al., 2020, Ameer et al., 2022).

Traditional mental health practices heavily rely on subjective patient reports regarding cognitive and emotional states, leading to decisions based on incomplete impressions. This reliance on superficial assessments may hinder accurate diagnoses and optimal treatment. Psychiatric disorders, characterized by dynamic gene-environment interactions across biological networks, form a continuous spectrum, complicating the clinical landscape. The intrinsic clinical diversity requires challenging trial-and-error methods to select treatments, potentially worsening conditions, and produce less-than-ideal results. Recognizing the limitations of current methodologies, there is a growing consensus on the imperative need for innovative artificial intelligence (AI) approaches to advance the field.

The human brain acts analogously, whereas the machine functions digitally. Humans learn from diverse experiences supported by motivation and logic. The human brain possesses significantly greater thinking capacity and problem-solving skills, enabling adaptation to the core of a situation without being confined to a specific pattern. By learning from vast datasets and recognizing patterns akin to human cognition, AI presents a paradigm shift in psychiatric research and precision medicine (Richards et al., 2022). AI's potential to efficiently solve problems and its adeptness at processing complex, heterogeneous, and multidimensional data positions it as a revolutionary tool in understanding and treating endotype-specific psychiatric disorders. Recent advancements in AI applications for psychiatric research and diagnosis underscore the transformative impact of integrating artificial intelligence into mental health practices (Bzdok and Meyer-Lindenberg, 2018, Esteva et al., 2019).

Psychiatric disorders have now become one of the critical public health challenges. There are many different types, with some of them having high prevalence. According to the global burden of disease assessment, these disorders account for 4.9 % of global disability-adjusted life-years (DALYs), with an age-standardized rate of 1566.2 per 100,000 people (Sun et al., 2023). Anxiety, depression, stress, and mood swings are the most common disorders. An accurate and early diagnosis can improve a patient’s quality of life. In recent research, it is observed that symptom-focused diagnosis is limited only to symptom relief treatment. As a result, for correct diagnosis, several data-driven techniques should also be employed to explore these illnesses' biological/neural mechanisms. The advancement in computational science has broadened the extent of evidence for mental healthcare. The use of artificial intelligence techniques like ML (machine learning) and DL (deep learning) (Fareed et al., 2022, Mahmood et al., 2022, Ahmed et al., 2022, Carcagn\`\i et al., 2023, Mora-Rubio et al., 2023, Imani, 2023, Ramzan and Dawn, 2023, Masuda and Yairi, 2023, Mahum et al., 2021) has increased. ML assesses the performance of test data and provides high-level results of clinical diagnosis (Park et al., 2021). The brain's structure is very complicated. EEG works on many individual neurons, each interacting with a neighboring neuron.

The most challenging part of brain-machine interaction is to infer emotional state from patterns and behaviors of electrical brain activity (Bird et al., 2019). Bioinspired classifiers and deep evolutionary optimization approaches have been used to classify emotional state and mental attention. Bird et al. generated the initial EEG dataset and selected some attributes. The neural network was optimized and then used for classification. Each step was done through biologically inspired computing. Evolutionary optimization was done by choosing discriminative attributes from the dataset for optimized classification. A new dataset was generated from selected attributes, and LSTM (long short-term memory) and MLP (multilayer perceptron) models were developed. Emotional identification should include physiological factors such as pupil dilation, skin conductance, the individual's heart rate, brainwave signals, facial expressions, and speech. The EEG is only a physiological reaction; how the emotion is felt cognitively is not very clear (Bos et al., 2006). To record the electrical activity of the brain, the electrode positioning is also very significant (Seal et al., 2020, Cimtay and Ekmekcioglu, 2020).

A voltage change occurs when a neuron in the human brain changes its state. Incoming signals trigger sodium ions into the cell, which causes a voltage rise. An action potential, also known as electric discharge, is triggered inside the brain when this voltage rise increases a certain threshold. This electric discharge travels down to other neighboring neurons. The voltage change event lasts for only two milliseconds inside neurons. During this voltage change, the voltage goes from −60 mV resting potential to +20 mV active potential (Brienza and Mecarelli, 2019, Tivadar and Murray, 2019, Sasidharan and Dutta, 2021). The activity of neurons close to electrodes can be observed in EEG. Brain tissues and skull bones distort the brain's electrical activity measured by EEG. That’s one of the main reasons EEG amplitude is in microvolts. The limbic structure inside the brain stem is responsible for emotional reactions. The hypothalamus inside the brain structure processes the incoming signals and triggers visceral physiological effects like galvanic skin response or increased heart rate (Bos et al., 2006).

Ghosh-Dastidar et al (Ghosh-Dastidar and Adeli, 2007). used the following three training algorithms, SpikeProp, QuickProp, and RProp, for epileptic seizure detection through EEG. Classification accuracy, computational efficiency, and number of convergence epochs were the measures of performance investigated for each algorithm. An extensive parametric analysis was performed to identify optimum parameter values and heuristic rules. Soleymani et al (Soleymani et al., 2015). detected and labeled expressions and physiological responses. Valence, arousal, and dominance (VAD) are emotion's most famous dimensional representations. Emotions need to be detected both from EEG signals and facial expressions. EEG signals and facial expressions of multiple subjects were analyzed and some emotional features like facial landmarks and power spectral density (PSD) from EEG were extracted. Different regression models were applied for valance detection. The relationship between facial expressions and EEG signals was identified through statistical analyses. In another study (Zhang and Lee, 2008), Qing Zhang identified emotions through natural scene images. Feature selection is a difficult task in emotion recognition systems. Functional magnetic resonance imaging (fMRI) gives images of those regions where emotional activity occurs. Using fMRI and EEG, emotional information was analyzed, corresponding visual features were extracted, and image emotions by visual stimulus were classified. GIST (General Image structure) is used to extract features of natural scenes.

In the current study, we have also worked on classifying different psychological disorders detection through EEG data using different DL models. The dataset used for psychiatric disorder classification was taken from Kaggle (Shadhin et al., 2020, https://www.kaggle.com/datasets/shashwatwork/eeg-psychiatric-disorders-dataset, 2023), consists of patients with six main disorders (addictive disorder, anxiety, mood disorder, obsessive-compulsive, schizophrenia, trauma & stress) along with their specific disorders (alcohol use disorder, acute stress, depression, behavioral addiction, panic, social anxiety, posttraumatic disorder, adjustment, and bipolar disorder). We used DL models (i.e., ANN (Agatonovic-Kustrin and Beresford, 2000), KNN (Guo et al., 2003), LSTM (Staudemeyer and Morris, 2019), Bi LSTM (Huang et al., 2015) and CNN-LSTM (Wang et al., 2016)) for the classification of main and specific disorders. Functional connectivity and spectral power features are the EEG parameters used. The primary objective of this study was to deepen our comprehension of psychiatric disorders by conducting a thorough analysis of neurophysiological signals, with a specific focus on EEG data. By employing sophisticated deep learning models and integrating both PSD and FC features across various frequency bands, the study aims to enhance the accuracy of psychiatric disorder classification. Including specific disorders within the main categories and exploring diverse deep-learning architectures contribute to a detailed investigation. Ultimately, this research seeks to provide valuable insights for more accurate and early diagnosis of psychiatric disorders, thereby improving patient outcomes in mental healthcare.

## Materials & methods

### Study design

**Fig. 1:**
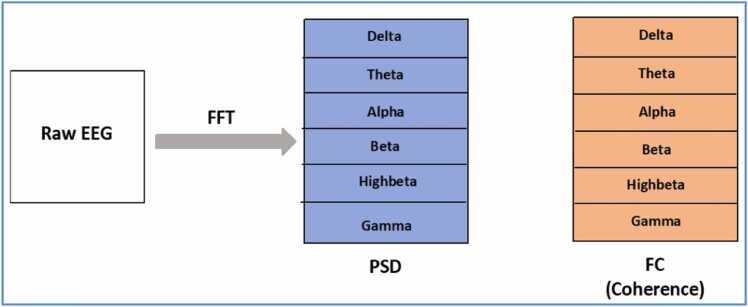
Representation of EEG signals into PSD (power spectral density) and FC (functional connectivity) features.

The study is a retrospective analysis that utilized a dataset obtained from Kaggle (Park et al., 2021). The data was collected and evaluated from patients diagnosed with six major categories of mental diseases, each of which was further classified into particular disorders. The primary objective of this study was to investigate and classify psychiatric disorders using EEG signals by applying DL models. Fig. 1 depicts a visual representation of the study design, feature selection, and extraction.

### Dataset

The dataset contained 945 subjects, of which 95 were healthy individuals. The age of all subjects was between 18 and 70 years. A single dataset is used in this study for the classification of Main & Specific Disorders. The EEG data was collected from individuals who had six types of main mental disorders: (Park et al., 2021), i.e., schizophrenia, mood disorders, anxiety, obsessive-compulsive disorders, addictive disorders, trauma, and stress-related disorders. Each main disorder was further categorized into specific disorders. Mood disorder is further classified into depression and bipolar disorder. Similarly, addictive disorder was categorized into alcohol use disorder and behavioral addiction. Trauma and stress-related disorders were further divided into three specific disorders: acute stress disorder, adjustment disorder, and posttraumatic stress disorder. Panic disorder and social anxiety disorder are particular types of anxiety disorder. EEG data also included a resting state of 5 minutes with eyes closed. The data attributes include age, IQ, sex, education, EEG date, and some EEG frequency values obtained from 19 channels. Each channel frequency values were further categorized into six frequency bands (delta, theta, alpha, beta, high beta, and gamma). Table 1 lists the EEG frequency ranging from 0.5 to 50 Hz*.*

**Table 1:** The frequency distribution of EEG signals.

**Name**	**Frequency Range**
Delta	0.5 – 4 Hz
Theta	4 – 8 Hz
Alpha	8 – 13 Hz
Beta	13 – 22 Hz
High beta	22 – 30 Hz
Gamma	30 – 50 Hz

The EEG dataset used in this work was taken from Kaggle (Park et al., 2021). The data defined by Park et al (Park et al., 2021)., includes all patients between 18 and 70 years of age diagnosed with any main disorder, which falls into nine specific disorders. The dataset also included patients with a medical history related to brain injury, neurodevelopmental disorder, or neurological disorder.

### EEG data parameters

EEG (Electroencephalogram) signals were chosen for the study because they provide a non-invasive way to measure and record electrical activity in the brain. Specifically, EEG signals are valuable for studying psychiatric disorders due to their ability to capture real-time electrical changes related to cognitive processes, emotions, and mental states. Abnormal patterns in EEG signals have been associated with conditions such as schizophrenia, mood disorders, anxiety, and more. By analyzing EEG data, researchers can better understand the neural mechanisms underlying psychiatric disorders, potentially leading to improved diagnostic methods and treatment strategies.

The data was acquired at a 500 – 1000 Hz sampling rate and 0.1 – 100 on-line filters through Neuroscan. FP1, FP2, F7, F3, Fz, F4, F8, T7, C3, Cz, C4, T8, P7, P3, Pz, P4, P8, O1, and O2 were the 19 channels selected for EEG data recording with a mastoid reference electrode (parameters for EEG signal capturing). The ground channel was between the Fz and FPz electrodes. EEG data were down-sampled to 128 Hz with electrode impedances below five kilo-ohms. Through the Neuroguide system, Fast Fourier transformation was used to convert EEG data into frequency domain with the following parameters: frequency range = 0.5 – 40 Hz, epochs = 2 s, sample rate = 128 samples/s, and resolution of 0.5 Hz with cosine taper window to minimize the leakage. The power spectral density (PSD)- an actual spectral power at the sensor level and functional connectivity (FC)- a synchronization measure between two signals represented as coherence, were used as EEG parameters. These parameters were calculated in delta, theta, alpha, beta, high-beta (25 – 30 Hz), and gamma frequency bands, as shown in Fig. 1. There were 19 PSD and 171 FC features. Both parameters were computed using all six frequency bands. The total number of features that could be used was (19 channels of PSD + 171 channels of FC) × six bands = 1140 features.

### Inclusion and exclusion criteria

Patients between the ages of 18 and 70 with primary diagnoses, classified into six main diagnoses and nine specific disorders, were included. This included schizophrenia, mood disorders, encompassing depressive disorder, bipolar disorders, anxiety, covering panic and social anxiety, obsessive-compulsive disorder, addictive disorders (involving alcohol use disorder and behavioral addiction, such as gambling and internet gaming), trauma and stress-related disorders, comprising post-traumatic stress disorder (PTSD), acute stress disorder, and adjustment disorder. Additionally, individuals were required to have no difficulty in reading, listening, writing, or understanding Hangeul (Park et al., 2021), and the data is available online at Kaggle (Park et al., 2021).

Cases of a lifetime or current medical history of neurological disorder or brain injury, neurodevelopmental disorder (e.g., intellectual disability [intelligence quotient (IQ) < 70], borderline intellectual functioning [70 < IQ < 80], tic disorder, attention deficit hyperactivity disorder), any other neurocognitive disorder, were excluded from the study (Park et al., 2021).

### Pre-Processing

First, all irrelevant columns (e.g., no., sex, EEG date, education, and IQ) and columns containing null values were filtered out of the dataset. For the binary classification of disorders, we applied one-hot encoding to the main disorder to generate binary vectors for each disorder (the same process was repeated for specific disorders). In these binary vectors, 0 and 1 indicate the absence or presence of a disorder in a given row. We then discretized the PSD and FC features to enable separate execution of the classification models on each feature set. The data was converted into a multidimensional array, where each row and its feature values were stored in a sub-array. Standardization was applied to normalize all feature values, with the mean and standard deviation calculated to scale the data. The dataset was split into 80 % for training and 20 % for testing, with a test size 0.2.

### Classification models

**Fig. 2:**
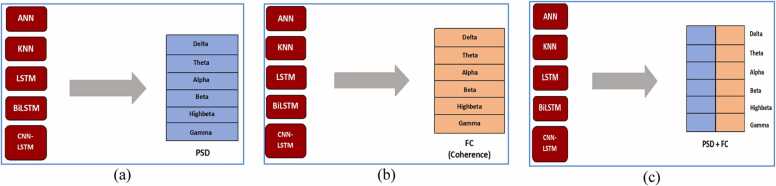
Representation of EEG signals into PSD (power spectral density) and FC (functional connectivity) features.

We used DL (deep learning) models (ANN, KNN, LSTM, Bi LSTM, and CNN-LSTM) to classify all psychiatric disorders. Each model was implemented with PSD and FC features separately against each frequency band. Each model's implementation involved utilizing PSD and FC features individually across each frequency band. Fig. 2**(a)** illustrates the implementation of all models with PSD features, while Fig. 2**(b)** showcases the implementation with FC features. Additionally, Fig. 2**(c)** depicts the implementation of all models incorporating both PSD and FC features.

### Artificial neural network (ANN)

ANN (Cruz et al., 2023) model can capture non-linear relationships between the target variable and input features. It can automatically learn related features from input data. The main reason for choosing this model is its ability to handle high-dimensional data, making it ideal for our dataset. In the ANN model architecture, first, we have the input layer with 64 neurons followed by two hidden layers with 32 and 16 neurons, respectively. ReLU activation function was used in input and hidden layers. For binary classification, there was only one neuron with a sigmoid activation function in the output layer. The input layer and each hidden layer(s) were followed by a dropout of 0.5.

### K-nearest neighbors (KNN)

The KNN (Khare et al., 2023) model considers K-nearest neighbors of data points and makes predictions. This allows the model to capture the data's decision boundaries and local patterns. This model directly stores the training data and uses it during prediction. This model can handle mixed data types without any explicit encoding. Outliers don’t have much influence in the decision-making process, as KNN relies on local information. In this KNN model, K-nearest neighbors were used as 9.

### Long short-term memory (LSTM)

LSTM stands for long short-term memory, similar to recurrent neural networks (RNNs). This model can learn long-term dependencies. LSTM (Xu et al., 2023, Sekhar et al., 2023) has memory cells that can store information selectively over time. LSTM works on the principle of three gates: input, forget, and output gate. The input gate determines the new information to store in memory. The forget gate discards some selected information from the previous hidden state, while the output gate decides which information should be given as the current hidden state from memory cells. We used 64 neurons as output in the first and a single neuron in the output layer with a sigmoid activation function.

### Bidirectional long short-term memory (Bi LSTM)

Bi LSTM (Sekhar et al., 2023) is an extended version of LSTM (Xu et al., 2023) architecture, which includes bidirectional processing (forward and backward). It captures past and future context against each time step, allowing the network to capture dependencies in both directions. There are separate sets of hidden states and weights for both directions of LSTM layers. The hidden forward and backward LSTM states are concatenated to get the final output. We utilized 64 neurons in the first layer of our Bi LSTM architecture, representing the forward LSTM layer, followed by a 32 neurons layer, representing the backward LSTM layer. The output layer contained one neuron with a sigmoid activation function.

### Convolutional neural network -long short-term memory (CNN-LSTM)

This is a hybrid model created by combining CNN (convolutional neural network) and LSTM (Imani, 2023, Ramzan and Dawn, 2023, Masuda and Yairi, 2023). First, the input data was passed to the convolutional layer. The CNN layer extracted the spatial features from the input data. Filters slide through input data to perform element-wise multiplications. The output from the CNN layer was passed to LSTM layers. The output from the LSTM layer was then passed through additional layers for classification. A convolutional 1D layer with 64 filters, a kernel size of 3, and a ReLU activation function was used after the LSTM layer. A 1D max-pooling layer with a pool size of 2 followed this. A dropout layer with a rate of 0.5 was then applied. Subsequently, another LSTM layer with 32 neurons was added. Finally, a dense layer with a single neuron and a sigmoid activation function was used for classification.

## Experimentation & results

We separately implemented all five models, ANN, KNN, LSTM, Bi LSTM, and CNN-LSTM, on both main and specific disorders. We implemented the models with PSD and FC features separately. PSD and FC features were evaluated with each frequency band for all main and specific disorders. We implemented models with all features (PSD and FC) for the binary classification of each disorder. To ensure authentic validation, test sets were separated before training the models. Furthermore, cross-validation was applied to each model to verify the results, and each model was trained on 100 epochs. The experimentation results on each feature are given below for each method exploited.

### Artificial neural network (ANN)

The binary classification of main disorders with PSD and binary classification through FC features of each band are represented in Table 2, Table 3, Table 4. This model used a Stochastic Gradient Descent (SGD) optimizer with Binary-Cross Entropy (BCE) loss as a hyperparameter. Table 2 represents the classification of main disorders (with both PSD and FC features) of all frequency bands. Table 3 shows that the ANN model performed more accurately against obsessive-compulsive disorder on PSD features of the gamma band with an accuracy of 97.35 %. The binary classification of specific disorders with PSD and FC are represented in Table 3 with all (PSD and FC) features, respectively. The highest accuracy of 97.35 % was achieved against social anxiety disorder and adjustment disorder on PSD features. In contrast, the highest accuracy was achieved against acute stress and social anxiety disorder on theta FC features, as shown in Table 4. Table 2 shows that the highest accuracy of 96.30 % was achieved through all features against adjustment disorder.

**Table 2:** Main & Specific disorder classification accuracy through ANN in percentage from all features.

**ANN Implementation on Entire Band**			
**Main Disorders**	**% Accuracy on Entire Band**	**Specific Disorders**	**% Accuracy on Entire Band**
Addictive disorder	78.31	Alcohol use disorder	88.36
Trauma and Stress	87.83	Acute stress disorder	95.24
Mood Disorder	65.08	Depressive disorder	75.13
**Obsessive Compulsive**	**96.83**	Behavioral addiction disorder	88.36
Schizophrenia	83.60	Panic disorder	93.12
Anxiety Disorder	87.30	Social anxiety Disorder	93.12
Addictive disorder	78.31	Posttraumatic stress disorder	94.71
Trauma and Stress	87.83	**Adjustment disorder**	**96.30**
Mood Disorder	65.08	Alcohol use disorder	88.36
		Bipolar disorder	92.59

*The entire band combines (PSD + FC) features.

**Table 3:** Classification accuracy in the percentage of Main disorders through each band's power spectral density (PSD) and functional connectivity (FC) features using ANN.

**ANN Implementation on PSD & FC Features of Main Disorder**							
**Main Disorder**		**Addictive disorder**	**Trauma and Stress**	**Mood Disorder**	**Obsessive Compulsive**	**Schizophrenia**	**Anxiety Disorder**
**Delta**	PSD	82.54	86.24	69.84	93.12	88.36	90.48
	FC	78.84	81.48	71.43	94.18	85.71	86.24
**Theta**	PSD	82.01	84.66	74.60	91.53	85.71	88.36
	FC	75.66	83.07	73.54	**95.24**	80.95	88.36
**Alpha**	PSD	84.66	88.89	72.49	94.18	84.66	83.07
	FC	81.48	88.89	72.49	94.18	84.66	83.07
**Beta**	PSD	78.84	84.66	73.02	92.59	87.30	89.42
	FC	78.31	84.66	62.96	93.65	86.77	84.13
**High Beta**	PSD	79.89	90.48	75.66	94.71	91.53	90.48
	FC	83.07	82.01	74.60	94.71	91.53	88.36
**Gamma**	PSD	82.01	86.77	71.96	**97.35**	86.24	93.12
	FC	80.42	86.77	67.20	92.06	89.95	89.95
**Whole**	PSD	80.95	81.48	68.78	96.30	87.83	91.01
	FC	78.84	84.66	66.14	94.18	85.19	89.42

**Table 4:** Classification accuracy in the percentage of Specific disorders through each band's power spectral density (PSD) and functional connectivity (FC) features using ANN.

**ANN Implementation on PSD & FC Features of Specific Disorders**										
**Main Disorder**		**Alcohol use disorder**	**Acute stress disorder**	**Depressive disorder**	**Behavioral addiction disorder**	**Panic disorder**	**Social Anxiety disorder**	**Posttraumatic stress disorder**	**Adjustment disorder**	**Bipolar disorder**
**Delta**	PSD	91.01	96.30	75.66	88.89	91.01	95.77	93.65	**97.35**	90.48
	FC	91.53	96.83	75.66	88.89	93.65	95.24	93.12	96.30	91.01
**Theta**	PSD	92.06	96.30	79.37	90.48	94.18	**97.35**	96.30	95.77	89.95
	FC	88.39	**97.35**	77.78	91.01	92.06	**97.35**	95.24	96.30	91.35
**Alpha**	PSD	91.01	95.24	75.13	91.53	92.59	94.18	92.59	95.77	91.01
	FC	87.30	95.77	76.19	88.89	96.83	96.30	93.65	94.18	94.71
**Beta**	PSD	88.36	93.13	75.13	88.89	93.12	94.71	94.71	94.18	93.65
	FC	90.48	**94.18**	70.90	90.48	92.59	95.24	94.71	96.30	95.77
**High Beta**	PSD	92.06	96.30	82.54	90.48	92.59	**97.35**	95.24	94.71	92.59
	FC	91.01	95.77	76.19	93.12	93.12	96.30	94.18	95.77	93.65
**Gamma**	PSD	90.48	94.71	80.42	91.01	92.59	93.12	92.59	96.30	94.18
	FC	87.30	96.83	77.25	90.48	95.24	94.18	94.71	93.65	92.06
**Whole**	PSD	85.71	95.77	75.66	89.95	93.65	93.65	94.18	94.71	92.06
	FC	89.95	95.24	78.84	88.36	93.12	96.30	96.83	96.30	94.18

### K-nearest neighbors (KNN)

The KNN implementation results for main disorders (with PSD and FC features) achieved the highest accuracy of 97.88 % against obsessive-compulsive main disorder on PSD features of the high beta band. In comparison, the highest accuracy of 97.35 % was achieved against obsessive-compulsive disorder on FC features of the gamma band, as depicted in Table 5.

**Table 5:** Classification accuracy in the percentage of Main disorders through each band's power spectral density (PSD) and functional connectivity (FC) features using KNN.

**KNN Implementation on PSD & FC Features of Main Disorder**							
**Main Disorder**		**Addictive disorder**	**Trauma and Stress**	**Mood Disorder**	**Obsessive Compulsive**	**Schizophrenia**	**Anxiety Disorder**
**Delta**	**PSD**	77.24	86.24	96.84	96.27	87.30	91.53
	**FC**	78.30	82.53	66.66	96.82	89.94	87.83
**Theta**	**PSD**	76.71	83.06	70.89	96.82	88.35	85.18
	**FC**	83.06	83.06	63.49	95.23	87.30	89.41
**Alpha**	**PSD**	80.42	82.53	65.60	95.76	87.30	91
	**FC**	82.53	92.59	65.60	94.70	88.88	91.53
**Beta**	**PSD**	78.83	87.83	70.89	95.76	84.65	90.47
	**FC**	73.54	86.24	96.31	93.65	84.65	88.88
**High Beta**	**PSD**	72.84	88.88	65.07	**97.88**	89.41	84.65
	**FC**	77.24	84.65	67.19	95.23	89.41	88.35
**Gamma**	**PSD**	79.89	83.06	66.66	95.23	87.83	88.88
	**FC**	79.89	85.18	69.31	**97.35**	86.77	90.47
**Whole**	**PSD**	79.36	84.12	68.25	94.17	86.24	92.06
	**FC**	80.95	88.35	66.66	94.17	85.18	89.41

Main disorder classification through all features is indicated in Table 2 - the highest accuracy of 94.70 % was achieved against obsessive-compulsive. KNN implementation on specific disorders (with PSD and FC features) got the highest accuracy of 97.35 % against acute stress disorder on PSD features of high beta band and shows the highest classification accuracy of 98.94 % for acute stress disorder through FC features of gamma band, as shown in Table 6. The results of specific disorders with all features are shown in Table 7. The highest accuracy of 96.29 % was achieved against panic disorder.

**Table 6:** Classification accuracy in the percentage of Specific disorders through each band's power spectral density (PSD) and functional connectivity (FC) features using KNN.

**KNN Implementation on PSD & FC Features of Specific Disorders**										
**Main Disorder**		**Alcohol use disorder**	**Acute stress disorder**	**Depressive disorder**	**Behavioral addiction disorder**	**Panic disorder**	**Social Anxiety disorder**	**Posttraumatic stress disorder**	**Adjustment disorder**	**Bipolar disorder**
**Delta**	PSD	87.83	94.70	78.83	93.12	94.17	96.29	92.06	94.17	89.94
	FC	86.65	93.65	82.53	87.83	93.65	95.76	96.82	95.23	94.70
**Theta**	PSD	88.88	93.12	75.66	91	93.65	92.06	95.76	96.29	92.59
	FC	92.59	94.17	75.13	88.35	94.70	94.70	97.35	95.76	92.59
**Alpha**	PSD	88.88	97.35	79.89	91	92.59	95.23	94.70	95.23	92.59
	FC	92.59	96.29	79.36	91.53	93.12	92.59	94.17	96.82	92.59
**Beta**	PSD	87.30	91.53	76.71	90.47	93.65	92.06	95.23	95.23	95.23
	FC	87.83	95.76	72.48	88.88	94.17	94.17	96.29	95.23	95.23
**High Beta**	PSD	90.47	97.35	80.42	90.47	91.53	94.17	94.70	94.70	89.41
	FC	88.88	95.23	77.24	89.94	93.12	92.59	95.23	95.23	89.94
**Gamma**	PSD	87.30	94.70	74.60	90.47	92.06	94.70	94.17	96.82	94.17
	FC	87.83	**98.94**	78.83	91	94.70	94.70	96.29	97.88	94.17
**Whole**	PSD	91	95.76	78.30	89.47	95.23	96.29	96.29	95.76	91
	FC	89.41	97.35	76.71	89.41	93.65	97.35	93.12	94.17	94.70

**Table 7:** Main & Specific disorder classification accuracy through KNN in percentage from all features.

**KNN Implementation on Entire Band**			
**Main Disorders**	**% Accuracy on Entire Band**	**Specific Disorders**	**% Accuracy on Entire Band**
Addictive disorder	79.36	Alcohol use disorder	87.83
Trauma and Stress	86.24	Acute stress disorder	95.23
Mood Disorder	68.78	Depressive disorder	76.71
**Obsessive Compulsive**	**94.70**	Behavioral addiction disorder	93.12
Schizophrenia	84.12	**Panic disorder**	**96.29**
Anxiety Disorder	88.88	Social anxiety Disorder	94.70
Addictive disorder	79.36	Posttraumatic stress disorder	94.17
Trauma and Stress	86.24	Adjustment disorder	95.76
Mood Disorder	68.78	Bipolar disorder	93.65
		Alcohol use disorder	87.83

### Long short-term memory (LSTM)

The LSTM implementation results for the main disorder (with PSD and FC features) are presented in Table 8. From the experimentation, it was observed that the highest accuracy of 96.83 % was achieved against obsessive-compulsive disorder on PSD features of the delta band. In this experiment, the Adam optimizer was used with a learning rate of 0.0001 to optimize the model. The highest accuracy of 96.83 % was achieved for obsessive-compulsive disorder on FC features of the alpha band. LSTM main disorder classification with all features is shown in Table 9. 93.65 % was the highest accuracy achieved against obsessive-compulsive disorder. For specific disorder classification, the highest accuracy of 98.94 % was achieved against acute stress disorder and adjustment disorder on PSD features. For specific disorder classification on FC features, the highest accuracy of 98.94 % was observed against social anxiety disorder. From Table 10, the PSD and FC feature experimentation of specific disorders can be looked at. Table 9 shows specific disorder classification on all features in which the highest accuracy of 96.30 % was observed against adjustment disorder.

**Table 8:** Classification accuracy in the percentage of Main disorders through each band's power spectral density (PSD) and functional connectivity (FC) features using LSTM.

**LSTM Implementation on PSD & FC Features of Main Disorder**							
**Main Disorder**		**Addictive disorder**	**Trauma and Stress**	**Mood Disorder**	**Obsessive Compulsive**	**Schizophrenia**	**Anxiety Disorder**
**Delta**	**PSD**	84.13	86.24	65.61	**96.83**	86.77	85.71
	**FC**	79.89	87.83	69.84	95.24	85.19	91.53
**Theta**	**PSD**	79.37	88.36	72.49	95.24	88.36	90.48
	**FC**	80.42	89.95	72.49	95.77	89.95	89.95
**Alpha**	**PSD**	81.48	89.95	76.19	93.65	80.95	88.36
	**FC**	79.89	86.24	73.54	96.83	85.71	87.3
**Beta**	**PSD**	76.72	86.24	72.49	95.77	85.71	86.77
	**FC**	83.6	89.95	69.84	93.12	86.24	88.36
**High Beta**	**PSD**	79.89	87.83	72.49	96.30	89.42	87.83
	**FC**	83.07	86.77	71.43	94.18	90.48	84.66
**Gamma**	**PSD**	77.25	85.71	74.07	94.71	83.07	88.89
	**FC**	79.37	88.89	73.02	95.24	89.42	91.01
**Whole**	**PSD**	78.84	87.83	71.96	95.24	92.59	89.95
	**FC**	76.19	89.95	67.72	94.18	88.89	88.89

**Table 9:** Main & Specific disorder classification accuracy through LSTM in percentage from all features.

**LSTM Implementation on Entire Band**			
**Main Disorders**	**% Accuracy on Entire Band**	**Specific Disorders**	**% Accuracy on Entire Band**
Addictive disorder	77.78	Alcohol use disorder	89.42
Trauma and Stress	86.77	Acute stress disorder	95.24
Mood Disorder	77.25	Depressive disorder	80.95
**Obsessive Compulsive**	**93.65**	Behavioral addiction disorder	91.01
Schizophrenia	84.66	Panic disorder	92.59
Anxiety Disorder	91.53	Social anxiety Disorder	94.18
Addictive disorder	77.78	Posttraumatic stress disorder	93.65
Trauma and Stress	86.77	**Adjustment disorder**	**96.30**
Mood Disorder	77.25	Bipolar disorder	93.12
		Alcohol use disorder	89.42

**Table 10:** Classification accuracy in the percentage of Specific disorders through each band's power spectral density (PSD) and functional connectivity (FC) features using LSTM.

**LSTM Implementation on PSD & FC Features of Specific Disorders**										
**Main Disorder**		**Alcohol use disorder**	**Acute stress disorder**	**Depressive disorder**	**Behavioral addiction disorder**	**Panic disorder**	**Social Anxiety disorder**	**Posttraumatic stress disorder**	**Adjustment disorder**	**Bipolar disorder**
**Delta**	PSD	89.42	95.77	82.01	90.48	91.53	94.71	95.24	96.83	91.01
	FC	86.24	96.83	78.31	77.25	91.53	93.65	93.12	96.3	89.42
**Theta**	PSD	91.53	96.3	80.95	86.24	90.48	93.12	92.59	96.3	92.59
	FC	90.48	96.3	75.13	76.72	94.71	96.3	95.77	97.35	92.06
**Alpha**	PSD	89.95	95.77	77.25	88.36	97.35	94.71	94.18	**98.94**	93.12
	FC	91.53	96.83	75.66	77.78	93.65	95.24	94.18	97.35	93.12
**Beta**	PSD	91.53	96.83	76.72	90.48	93.65	95.24	96.83	96.3	91.53
	FC	89.95	95.77	78.31	78.31	96.83	**97.88**	93.65	97.35	92.06
**High Beta**	PSD	90.48	94.18	79.37	91.01	93.65	93.65	92.06	93.12	91.53
	FC	89.42	97.35	82.54	80.42	92.06	94.18	94.18	97.35	92.59
**Gamma**	PSD	87.83	96.83	83.6	89.95	95.77	96.3	95.24	96.83	96.3
	FC	89.42	93.65	76.72	81.48	93.65	95.77	91.53	95.24	92.06
**Whole**	PSD	90.48	**98.94**	75.13	91.01	94.18	94.18	94.71	95.24	92.59
	FC	94.18	95.77	79.89	92.06	94.18	94.71	96.83	95.77	92.59

### Bidirectional long short-term memory (Bi LSTM)

The experimentation results (listed in Table 11) of Bi LSTM against all main disorders with PSD features show the highest accuracy of 96.83 % was achieved against obsessive-compulsive disorder. The observation from the experimentation was that obsessive-compulsive disorder is evaluated more accurately on FC features of the delta band with an accuracy of 97.88 %. Adam optimizer was used in this experiment with a learning rate of 0.001. Table 12 shows the main disorder classification through all features, and the highest accuracy of 93.12 % was achieved against obsessive-compulsive disorder. Bi-LSTM implementation results are represented in Table 13 for binary classification of specific disorders on PSD features of all frequency bands, in which the highest accuracy of 98.94 % was achieved against adjustment disorder through beta band and the highest accuracy for acute stress disorder on FC features of delta band. Table 12 shows the highest accuracy of adjustment disorder (with PSD and FC features of all frequency bands), in which the highest accuracy of 96.83 % was achieved for adjustment disorder.

**Table 11:** Classification accuracy in the percentage of Main disorders through each band's power spectral density (PSD) and functional connectivity (FC) features using Bi-LSTM.

**Bi-LSTM Implementation on PSD & FC Features of Main Disorder**							
**Main Disorder**		**Addictive disorder**	**Trauma and Stress**	**Mood Disorder**	**Obsessive Compulsive**	**Schizophrenia**	**Anxiety Disorder**
**Delta**	**PSD**	80.42	84.13	66.14	95.77	88.89	90.48
	**FC**	78.31	86.24	71.96	**97.88**	89.42	90.48
**Theta**	**PSD**	81.48	86.24	70.37	94.71	90.48	86.77
	**FC**	79.89	87.3	69.31	95.77	91.01	91.01
**Alpha**	**PSD**	78.84	88.89	69.31	**96.83**	86.24	88.36
	**FC**	80.42	86.77	69.31	93.12	90.48	88.36
**Beta**	**PSD**	75.66	86.24	67.72	93.65	84.66	86.24
	**FC**	83.6	88.89	70.37	96.83	85.71	89.95
**High Beta**	**PSD**	79.37	86.24	72.49	96.3	87.3	91.53
	**FC**	82.54	85.19	70.37	94.71	84.13	88.36
**Gamma**	**PSD**	82.01	87.83	75.13	**96.83**	85.19	87.3
	**FC**	80.42	86.24	68.25	96.3	85.71	84.13
**Whole**	**PSD**	80.95	85.71	67.72	94.18	86.24	87.83
	**FC**	81.48	87.3	71.43	95.77	87.3	88.89

**Table 12:** Main & Specific disorder classification accuracy through Bi-LSTM in percentage from all features.

**Bi-LSTM Implementation on Entire Band**			
**Main Disorders**	**% Accuracy on Entire Band**	**Specific Disorders**	**% Accuracy on Entire Band**
Addictive disorder	81.48	Alcohol use disorder	89.95
Trauma and Stress	86.24	Acute stress disorder	96.30
Mood Disorder	73.02	Depressive disorder	77.25
**Obsessive Compulsive**	**93.12**	Behavioral addiction disorder	94.18
Schizophrenia	88.36	Panic disorder	94.71
Anxiety Disorder	86.77	Social anxiety Disorder	95.77
Addictive disorder	81.48	Posttraumatic stress disorder	90.48
Trauma and Stress	86.24	**Adjustment disorder**	**96.83**
Mood Disorder	73.02	Bipolar disorder	91.01
		Alcohol use disorder	89.95

**Table 13:** Classification accuracy in the percentage of Specific disorders through each band's power spectral density (PSD) and functional connectivity (FC) features using Bi-LSTM.

**Bi-LSTM Implementation on PSD & FC Features of Specific Disorders**										
**Main Disorder**		**Alcohol use disorder**	**Acute stress disorder**	**Depressive disorder**	**Behavioral addiction disorder**	**Panic disorder**	**Social Anxiety disorder**	**Posttraumatic stress disorder**	**Adjustment disorder**	**Bipolar disorder**
**Delta**	PSD	91.53	95.24	80.95	90.48	91.01	93.12	91.53	94.71	92.59
	FC	88.89	**98.94**	80.95	88.89	92.06	93.12	95.24	96.3	94.18
**Theta**	PSD	92.59	95.77	80.42	93.12	95.77	95.24	92.06	92.59	93.65
	FC	87.83	96.3	80.95	88.36	94.18	97.35	92.06	96.83	88.89
**Alpha**	PSD	89.42	93.65	82.54	90.48	92.06	97.35	93.12	96.3	92.59
	FC	90.48	96.83	78.84	89.95	93.65	94.18	95.77	95.24	93.65
**Beta**	PSD	88.36	97.35	70.9	91.01	96.3	94.71	94.71	**98.94**	90.48
	FC	88.89	95.24	81.48	89.42	93.65	97.35	95.24	96.83	92.06
**High Beta**	PSD	89.42	97.88	78.31	91.53	91.01	94.18	93.65	96.3	92.59
	FC	88.36	96.3	76.72	92.06	91.53	94.71	94.18	96.83	94.18
**Gamma**	PSD	91.53	93.65	81.48	90.48	92.59	95.24	96.3	96.83	96.83
	FC	86.24	97.35	77.78	91.01	91.53	95.24	92.06	97.35	89.95
**Whole**	PSD	86.24	96.83	76.72	87.3	95.24	95.24	95.24	96.3	93.65
	FC	88.36	96.83	80.95	90.48	95.24	95.77	95.77	96.83	92.59

### Convolutional neural network -long short-term memory (CNN-LSTM)

Classification results of main disorders through CNN-LSTM (with PSD and FC features) are shown in Table 14. The highest accuracy of 97.35 % for obsessive-compulsive disorders on PSD features of the theta band is observed. In this experiment, we used Adam optimizer to reach the global minima. The highest accuracy of 96.83 % was seen for obsessive-compulsive disorder on FC features of the delta band. Table 15 shows the classification of main disorders on all features (PSD and FC both), and 92.06 % was the highest accuracy achieved for obsessive-compulsive disorder. Furthermore, it shows the classification results of specific disorders on all features, and the highest accuracy of 96.83 % was achieved against adjustment disorder.

**Table 14:** Classification accuracy in the percentage of Main disorders through each band's power spectral density (PSD) and functional connectivity (FC) features using CNN-LSTM.

**CNN-LSTM Implementation on PSD & FC Features of Main Disorder**							
**Main Disorder**		**Addictive disorder**	**Trauma and Stress**	**Mood Disorder**	**Obsessive Compulsive**	**Schizophrenia**	**Anxiety Disorder**
**Delta**	**PSD**	79.89	82.54	75.66	96.83	90.48	89.95
	**FC**	78.84	89.42	67.72	**96.83**	87.83	87.3
**Theta**	**PSD**	79.37	85.71	76.19	**97.35**	87.83	88.36
	**FC**	81.48	84.66	72.49	95.77	86.77	86.77
**Alpha**	**PSD**	76.19	86.77	70.9	94.18	84.66	91.53
	**FC**	80.42	80.42	70.37	95.77	87.83	88.89
**Beta**	**PSD**	78.31	84.13	74.07	95.77	86.77	89.95
	**FC**	81.48	85.71	70.9	94.71	92.59	88.89
**High Beta**	**PSD**	82.54	87.83	73.02	96.3	85.19	91.01
	**FC**	75.66	88.89	72.49	95.77	87.83	85.71
**Gamma**	**PSD**	84.66	87.3	75.66	96.83	87.83	89.42
	**FC**	80.95	86.77	70.9	94.71	86.77	92.06
**Whole**	**PSD**	80.42	87.83	67.2	90.48	88.36	87.83
	**FC**	82.54	86.77	72.49	96.3	85.71	89.42

**Table 15:** Main & Specific disorder classification accuracy through CNN-LSTM in percentage from all features.

**CNN-LSTM Implementation on Entire Band**			
**Main Disorders**	**% Accuracy on Entire Band**	**Specific Disorders**	**% Accuracy on Entire Band**
Addictive disorder	82.01	Alcohol use disorder	90.48
Trauma and Stress	86.77	Acute stress disorder	96.30
Mood Disorder	70.37	Depressive disorder	77.78
**Obsessive Compulsive**	**92.06**	Behavioral addiction disorder	92.06
Schizophrenia	86.24	Panic disorder	89.42
Anxiety Disorder	88.36	Social anxiety Disorder	95.77
Addictive disorder	82.01	Posttraumatic stress disorder	95.77
Trauma and Stress	86.77	**Adjustment disorder**	**96.83**
Mood Disorder	70.37	Bipolar disorder	93.65
		Alcohol use disorder	90.48

### Comparison Analysis

**Fig. 3:**
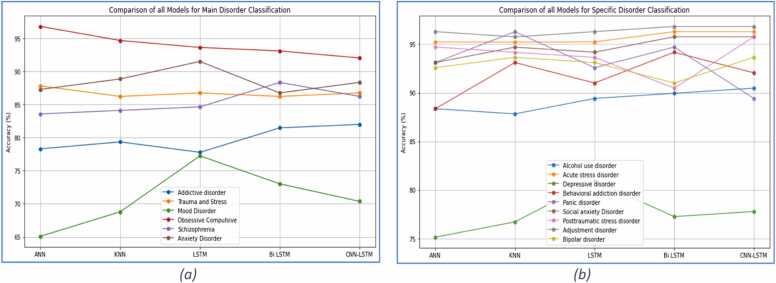
Classification of Main and Specific disorders through all models including all features.

To examine which model fared best against any main or specific disorder, this section presents some of the best results of all models acquired through all features of the full band. Fig. 3(a) shows that the highest accuracy was achieved through the ANN model against obsessive-compulsive as the main disorder. Fig. 3(b) represents the comparison analysis of all models against specific disorders. It can be concluded that the highest accuracy was achieved through Bi-LSTM and CNN-LSTM against adjustment disorder.

## Discussion

Psychiatric disorders are one of the most common mental health issues people are facing nowadays. A lot of workload at a job causes pressure on the brain, which results in stress, or whenever a person faces a challenging situation, he feels some stress or panic. Sometimes, people are not comfortable sharing everything that is going on in their minds, and due to a lack of information, doctors cannot identify what kind of mental issue the person is facing. To overcome this issue, MRI images were also used to get a proper diagnosis of mental disorders. Due to the high cost of image data, EEG signal is a better and cost-effective choice to record brain activity for the detection of mental disorders and epilepsy. In the current work, we have also used EEG signals for the detection of different psychiatric disorders through DL models. Prior works (Bos et al., 2006, Seal et al., 2020, Cimtay and Ekmekcioglu, 2020, Ghosh-Dastidar and Adeli, 2007, Soleymani et al., 2015, Zhang and Lee, 2008) mostly exploited ML models, but besides these, we improved the accuracy by incorporating DL models and techniques. First, we removed all the irrelevant columns from our dataset and then we converted all psychiatric disorders into binary vectors through one hot encoding. We implemented classification models on PSD and FC features separately. The classification models used for this work are ANN, KNN, LSTM, Bi LSTM, and CNN-LSTM.

In this study, we worked on psychiatric disorder classification through the lens of advanced DL models applied to EEG signal analysis. The robust methodology employed, which includes the utilization of a comprehensive dataset featuring 850 subjects diagnosed with diverse psychiatric disorders, underscores the significance of the findings. The inclusion criteria were age, specific diagnoses, and language proficiency, which enhanced the study's precision in characterizing the targeted population. The focal point of our investigation lies in the efficacy of DL models. Our exploration into PSD and FC parameters across various frequency bands contributes to a nuanced understanding of EEG signals and their relevance in psychiatric disorder classification. The novelty lies in integrating PSD and FC features across multiple frequency bands, encompassing delta, theta, alpha, beta, high beta, and gamma, to enhance the accuracy of psychiatric disorder detection.

This research yields promising results in classifying psychiatric disorders using EEG data and diverse deep-learning models. Notably, integrating PSD and FC features across multiple frequency bands enhances the accuracy of disorder detection. The study demonstrates that the ANN model achieved 96.83 % in identifying obsessive-compulsive disorder with entire (PSD + FC) band features. In comparison, both Bi LSTM and CNN-LSTM exhibit outstanding accuracy of 96.83 % in classifying adjustment disorder among specific disorders with entire band results. The multi-frequency band analysis and inclusion of specific disorders contribute to a nuanced understanding of neurophysiological patterns associated with different psychiatric conditions. Overall, the research underscores the potential of advanced computational models for precise and early mental health diagnosis.

Shah et al (Shah et al., 2023). introduced a method using a deep neural network (DNN) called the Temporal Spatial Network (ETSNet) for a new dataset on psychiatric disorders. They achieved impressive outcomes in their research, but the accuracy for classifying resting state was 93.15 %, which may not be sufficient for individual test cases. Additionally, their method was quite complex and required significant computational resources due to using DNNs. On the other hand, Tasci et al (Tasci et al., 2023). explored the use of electroencephalography (EEG) to improve the diagnosis and monitoring of severe psychiatric disorders, namely intellectual disability, schizophrenia, and bipolar disorder. They collected EEG data from 69 subjects, creating two new feature extraction methods—quantum local binary patterns (QLBP)—to enhance traditional local binary pattern techniques. Their model demonstrated high classification accuracies for the mentioned mental health conditions by utilizing advanced feature selection methods and a KNN classifier with leave-one-subject-out cross-validation. These promising results suggest the strong potential of their QLBP method and EEG signals were effective in diagnosing and monitoring psychiatric disorders. The results on the schizophrenia and bipolar disorder accuracies were 94.36 % and 93.49 %, respectively.

Emre et al (Emre et al., 2023). investigated the potential of using EEG data as a biomarker for psychiatric diseases, traditionally diagnosed through symptom-based approaches like DSM (Diagnostic and Statistical Manual of Mental Disorders) and ICD (International Classification of Diseases), supplemented by patient reports and physician expertise. They analyzed a dataset of EEG measurements from 550 patients with various psychiatric disorders and 84 healthy individuals using ML methods to differentiate and classify these conditions. They utilized 5-fold cross-validation to optimize hyperparameters across different models, including random forest, SVM (support vector machine), and ANN. Their results demonstrated high accuracy in classifying disease groups, especially ADHD (attention deficit and hyperactivity disorder), depression, and schizophrenia, suggesting that EEG data could indeed serve as a reliable biomarker for psychiatric conditions.

## Conclusion & Recommendations

The study concluded that when analyzed with advanced DL models, EEG signals can serve as an effective diagnostic tool for psychiatric diseases. The models improved upon traditional diagnostic approaches, offering an efficient and cost-effective means of classifying psychiatric disorders. The highest accuracy of 98.94 % was achieved through KNN and LSTM with delta FC features and entire band PSD features against acute stress disorder. We used binary classification for psychiatric disorders classification. Future work should continue to refine these models and explore their implementation in clinical settings, potentially improving patient outcomes through earlier and more accurate diagnoses. These advancements in EEG analysis for psychiatric disorders could revolutionize the way mental health issues are detected and managed, making a substantial impact on the field of psychiatry.

## Limitations

Park et al (Park et al., 2021). introduced the data and it is available on Kaggle. The study, while promising, encounters several limitations that should be addressed in future research. Firstly, the EEG data was collected from a finite number of subjects, which may not represent the entire spectrum of psychiatric conditions. A larger, more diverse sample size could enhance the generalizability of the findings. Secondly, the complexity of psychiatric disorders, which often present comorbidities and symptom overlap, may not be fully captured by the EEG signals and the algorithms used. This could potentially limit the model's diagnostic precision. Thirdly, the study relied on ML models that, despite their high accuracy, may not account for the dynamic nature of psychiatric symptoms over time. Continuous monitoring and adaptive models could provide a more accurate representation of the disorders. Fourthly, the study's models were developed and tested in a controlled environment, which may not reflect real-world clinical settings. External validation with data from clinical practice is necessary to confirm the effectiveness of the proposed models. Finally, the cost-effectiveness and practicality of implementing such advanced EEG analysis and ML models in routine clinical practice were not assessed. Further studies should consider the scalability and integration of these technologies into existing healthcare systems.

## Ethical standards

I have read and have abided by the statement of ethical standards for manuscripts submitted to Neuroscience.

## CRediT authorship contribution statement

**Zaeem Ahmed:** Writing – review & editing, Writing – original draft, Visualization, Methodology, Investigation, Formal analysis. **Saman Shahid:** Writing – review & editing, Writing – original draft, Validation, Supervision, Resources, Conceptualization. **Aamir Wali:** Writing – original draft, Methodology, Investigation, Formal analysis, Data curation. **Tunc Asuroglu:** Writing – review & editing, Validation, Supervision. **Jawad Rasheed:** Writing – original draft, Visualization, Validation, Software, Resources, Investigation. **Shahid Zikria:** Writing – original draft, Validation, Software, Resources, Formal analysis.

## Declaration of Competing Interest

None.
